# Pilot trial of STAR in the medical ICU

**DOI:** 10.1186/cc10779

**Published:** 2012-03-20

**Authors:** LM Fisk, AJ Le Compte, GM Shaw, S Penning, T Desaive, JG Chase

**Affiliations:** 1 University of Canterbury, Christchurch, New Zealand; 2 Christchurch Hospital, Christchurch, New Zealand; 3 Université de Liege, Belgium

## Introduction

Medical ICU patients often develop stress-induced hyperglycemia. Regulating blood glucose (BG) levels in these patients using insulin can be difficult due to varying patient conditions and therapy, leading to increased risk of hypoglycaemia. This abstract describes a pilot trial of STAR, a computerised risk-management accurate glycemic control (AGC) protocol.

## Methods

Thirteen hyperglycaemic patients (BG >145 mg/dl) were consented from Christchurch Hospital ICU. The BG target range was 80 to 145 mg/dl or 108 to 162 mg/dl (chosen clinically). Model-based insulin sensitivity was calculated for every measurement and its variability for the next 1 to 3 hours forecast using stochastic models. These data and model were used to calculate new insulin/nutrition interventions for the next 1 to 3 hours, limiting risk of BG <80 mg/dl to a maximum of 5%. Nursing staff selected the BG measurement interval to manage workload. Insulin was delivered as boluses (max 6 U/hour; max increase +2U/intervention), with infusions up to 3 U/hour for highly resistant patients. Nutrition was 30 to 100% of clinical goal feed (max change ±30% per intervention) and constant rates were used when desired clinically. Limiting insulin/nutrition changes prevents over-response to erroneous BG measurements and results were resampled hourly for consistency. Approval was granted by the Upper South A Regional Ethics Committee (Christchurch, New Zealand).

## Results

Median BG was 109 mg/dl for 80 to 145 mg/dl target patients and 145 mg/dl for 108 to 162 mg/dl target patients. In total, 85.6% of time was in the specified target band, with 1.18% of BG <72 mg/dl and 2.41% BG <80 mg/dl. BG measurement frequency was 13.3 measures/day. Per-patient median carbohydrate intake was 10.7 g/hour (IQR: 4.0 to 11.9 g/hour) and median insulin usage was 2.5 U/hour (IQR: 1.75 to 3.5 U/hour). Requirements varied considerably by patient. Observed response to insulin varied by a factor of 14× between patients. Accurate control was maintained over a range of metabolic conditions, and STAR adapted safely to therapies including high-dose steroids, long-acting insulin (Glargine) and changing insulin response.

## Conclusion

STAR provided AGC in a clinical setting. Tight and accurate control was able to be extended to patients with a range of metabolic requirements, and the risk-management approach proved capable of balancing clinical workload and risks presented by patient variability.

